# Leveraging the potential of the German operating room benchmarking initiative for planning: A ready-to-use surgical process data set

**DOI:** 10.1007/s10729-024-09672-9

**Published:** 2024-05-02

**Authors:** Grigory Korzhenevich, Anne Zander

**Affiliations:** 1 Institute for Operations Research, Karlsruhe Institute of Technology, Karlsruhe, Germany; 2 Center for Healthcare Operations Improvement and Research, University of Twente, Enschede, The Netherlands

**Keywords:** Surgical Process Data, Data analysis, German Perioperative Procedural Time Glossary, Operating Room Benchmarking Initiative, Operating Room Planning, Operations Research

## Abstract

We present a freely available data set of surgical case mixes and surgery process duration distributions based on processed data from the German Operating Room Benchmarking initiative. This initiative collects surgical process data from over 320 German, Austrian, and Swiss hospitals. The data exhibits high levels of quantity, quality, standardization, and multi-dimensionality, making it especially valuable for operating room planning in Operations Research. We consider detailed steps of the perioperative process and group the data with respect to the hospital’s level of care, the surgery specialty, and the type of surgery patient. We compare case mixes for different subgroups and conclude that they differ significantly, demonstrating that it is necessary to test operating room planning methods in different settings, e.g., using data sets like ours. Further, we discuss limitations and future research directions. Finally, we encourage the extension and foundation of new operating room benchmarking initiatives and their usage for operating room planning.

## Highlights

- We show the suitability of the surgery process data (with high levels of quantity and quality, standardization, and multi-dimensionality) from the German Operating Room Benchmarking initiative for operating room planning.
- We present a processed data set of case mixes and detailed surgery process duration distributions grouped with respect to hospital level of care, surgical specialty, and type of surgical patient.
- We make the processed data set freely available for researchers working on operating room planning.
- We show the necessity of operating room planning methods to be tested on different realistic settings since, e.g., hospitals of different care levels exhibit significantly different case mixes.
- We show benefits for practitioners to join or set up new benchmarking initiatives.

## Introduction

The operating room (OR) plays a crucial role in a hospital’s operations since, for most hospitals, a significant fraction of treated patients and generated revenues are associated with surgical services [49]. Because of this and because an OR is typically a highly complex system with many different stakeholders, expensive resources, time-sensitive processes, and an inherently high level of uncertainty, optimizing the efficiency of OR operations through adequate planning is crucial.

Research on operating room planning in Operations Research is popular and extensive [14, 16, 31, 35, 38, 41, 70, 75, 94, 99]. To test modeling and solution approaches, input data is needed, which is the focus of our work. We believe that to compare different models and solution techniques, they should be tested on different data sets representing different OR settings. Here, so-called benchmarking sets can be used [53]. Benchmarking sets represent collections of instances for particular (optimization) problems [47]. They can be based on fictional (i.e., generated) or real-world data [53]. Since the research on OR planning is implementation-oriented [14], the real-world data approach is more desirable. To generate such benchmark sets, real data should be collected systematically and in a standardized manner. The latter aspect is crucial to enable comparisons across organizations and aggregation of multiple data sources if desired.

However, data collection costs regarding technical, organizational, and financial resources are high [55], while the purposes aside from mandatory legal compliance might not always be apparent to the decision-makers. Consequently, real-world data for the research on OR planning is still scarce. If real data sets are used, they are often small or low-dimensional. Typically, the data from only one hospital is used [38]. Thus, only this hospital’s specific OR context regarding organization, resources, surgical portfolio, and procedures is being investigated. In the face of the just-described scarcity of real-world OR data, it is remarkable that there is an OR benchmarking initiative in the case of German-speaking countries. This initiative has been around for almost 15 years. Over 320^1^ German, Austrian, and Swiss clinics record and submit their surgical data in a standardized way. The database of the benchmarking program contains millions of surgical records [9]. Each data point represents a performed surgery and includes data on different surgery-related parameters.

For the participating hospitals, the primary purposes of the benchmarking initiative are to compare their OR performance regarding particular KPIs such as OR utilization among each other and to evaluate the development of one’s performance over time [9]. However, we argue that the data collected for benchmarking purposes can also be used for scientific purposes and research on OR planning.

We find the data suitable for studies on OR planning for multiple reasons. In a nutshell, the data shows high levels of quantity and quality, standardization, and multi-dimensionality. Multiple process time stamps are recorded per surgery, which enables detailed modeling of the surgical process, i.e., by breaking a surgery down into several process steps. For our purpose, by “surgical process data”, we denote the data on surgical process steps durations and consider the entire perioperative process as the scope of this definition.

We argue that the surgical process data from the OR benchmarking initiative of German hospitals especially has the potential for detailed modeling approaches of the short-term (“operational”) [37] OR planning, i.e., surgery scheduling, in particular. However, it can also be used for studies on OR process design. Regarding the investigation approach, the highly detailed data seems most suitable for simulative approaches and Job-Shop-like models. We note that the data can be aggregated to a lower level of detail to be used as input for low-detailed types of planning models as well.

This study aimed to process a data set from the OR benchmarking initiative of German-speaking countries for research on OR planning for the first time and to make it ready for fellow researchers to use. For this, we used the benchmarking data from 2019 and derived different OR settings based on parameters such as hospital level of care (LOC) or surgical specialty. For each setting, we have calculated distributions of surgical process durations and case mixes of surgical procedures, representing the surgical portfolio of the respective OR setting. One particular focus of our study was to model a surgery, not in its entirety, but to distinguish several process steps and to view them separately so that the data could be used in detailed model approaches, as mentioned previously. Concrete benchmark sets and problem instances can be generated from our collection of surgical case mixes and process duration distributions. We discuss in detail how this could be approached and suggest several OR planning problems and investigation approaches for which such benchmark sets could be useful. The collection of case mixes and process duration distributions can be accessed freely online [48].

One purpose of our study is to justify the practical relevance of the systematic collection of surgical process data in the context of prospective OR planning and to encourage hospitals and OR managers to re-evaluate their current data collection practices. Joining many fellow researchers, we want to draw the practitioners’ attention to the potential of (data-based) OR planning methods from the field of Operations Research.

The paper is organized as follows: In Section 2, we present related literature on surgical process data and its use in practice and OR planning research, as well as on benchmark sets and their suitability for testing different modeling and solution approaches. We also list further existing international OR benchmarking initiatives, which might have a potential for scientific studies similar to the potential of the benchmarking program we describe here. In Section 3.1, we present the said OR benchmarking initiative of German-speaking countries in detail before we describe the data collected throughout the initiative and how we processed the *2019 data set* and present it in our data collection in Section 3.2. Section 3.3 discusses the general potential and benefits of the benchmarking data and specifically of our collection of surgical case mixes and process duration distributions for different OR settings. We finish with a detailed discussion on the limitations of the benchmarking data and our approach and suggest ways to address those issues. Section 4 presents a concluding summary of our work and an outlook for future research.

## Literature review

### Surgical process data in OR practice

In OR operations, collecting specific surgical process data can be mandatory for hospitals for quality assurance and accounting reasons, based on prevalent regulations [55]. While data collection standards might not always be mandatory, it is essential to ensure consistent documentation over time and valid benchmarking [7, 11]. The data on process durations is usually routinely collected during surgery as time stamps for particular process milestones, e.g., OR entry or incision [7, 11]. The data on surgery process durations are being used in practice for retrospective performance analysis [11] as well as for duration forecasting in prospective surgery planning. The latter represents its own widely elaborated research field in the literature [30]. Typically, specific parameters are identified as significant predictors for surgery duration, e.g., surgery type or operating surgeon [42, 86].

### Surgical process data in studies on OR planning

Several systematic reviews touch upon the use of surgical process data within the reviewed studies [14, 31, 35, 38]. However, we could not find any reviews that would give a comprehensive insight into this topic. We present a short summary of our literature findings.

Researchers either use real-world data to model surgical process durations or generate fictional problem instances [38]. The former is usually preferred to ensure better implementability of the model or algorithm [14]. In a model, the data is used for two purposes: To model the realized and the predicted process duration. Both can be done deterministically, e.g., by using actual recorded durations for the former [30] and calculating mean values from historical data for the latter [49, 58]. Typically, however, the realized process durations are modeled stochastically [14] by fitting distributions for the process durations from empirical data [99]. The predicted process durations can be modeled using the parameters of the fitted distributions [36] or, alternatively, by using linear regression [49] or other Machine Learning algorithms [30].

Surgical process data is typically either grouped by specific parameters within a study or chosen from an overall data set according to the scope of the study. This corresponds with the above research on potential predictors of surgical process durations [42]. For example, the differentiation by hospital or hospital type is mostly done implicitly, as many studies use data from one hospital. The same holds for surgical specialty unless several surgical departments are being considered simultaneously, e.g., for a Master Surgery Schedule construction [57] or joint surgery scheduling [36]. Surgery or patient characteristics can be used to break down the data further. For example, surgery urgency (elective vs. non-elective) [96] or type of surgical patient (inpatient vs. outpatient) [97], although again - many studies focus on one urgency or case type and choose the data from their overall data sets accordingly [34]. Surgery type can be used as a grouper based on the actual surgical procedure(s) [80] or own classifications [36, 44, 49, 54, 67, 76]. Other potential groupers are patient age or diagnosis [54]. Finally, data classification can also be done based on resources involved in the surgery process, i.e., staff members [49, 54, 62], operating rooms or medical equipment [69]. We do not give a comprehensive list here. The research on surgery duration prediction can be consulted for further possible grouping parameters.

The level of detail in surgical process data and the corresponding modeled surgical process differ among studies. We propose that this could be another interesting aspect for future systematic reviews. Many studies view a surgery as a whole and focus on the intraoperative phase [36, 89]. Although it is not always clear in this case what particular process milestones define the “case time,” most of the time, the wheels-in to wheels-out duration can be assumed [77]. Many studies additionally consider pre- and postoperative surgery phases [4, 33, 69] and the corresponding spatial resources such as the preoperative holding unit, post-anesthesia care unit (PACU), or intensive care unit [38]. Some studies model turnover (or cleaning or OR-setup) time separately [4, 5, 12, 30, 32, 49, 61, 67, 76]. Studies such as Batun et al. [5], Brown et al. [12], Holmgren and Persson [40], Kougias et al. [49], Messer et al. [61], Ozen et al. [67] model the actual process of a surgery with three main process steps: Pre-incision (takes place either in the OR, i.e., OR entry to incision [12, 49, 67], or in a separate preparation room [40, 61]), incision-to-closure and post-incision (i.e., closure to OR exit [12, 49, 67]). Batun et al. [5] and Ozen et al. [67] additionally model “surgeon turnover.” This process step starts immediately after closure and occurs parallel to post-incision and OR cleaning. As Messer et al. [61] are concerned with finding the optimal number of OR transfer rooms, they additionally model the inward transfer of the patient into the OR area before pre-incision and the outward transfer after post-incision. Latorre Núñez et al. [51] model the pre-incision phase in more detail and distinguish between four different preparation or setup steps: Patient, OR, surgeon, and further resources. Riise et al. [74] focus even more on the surgical resources by considering process steps such as “removal of any superfluous equipment from the operating room” or “removal of used equipment.” Note that especially for the intraoperative phase, i.e., the actual surgical intervention, an extremely high level of detail in process modeling can theoretically be achieved by identifying individual surgical manipulations [59, 64, 100]. Such a high level of detail can help estimate the (remaining) duration of a particular surgery [3, 100]. However, for the type of OR resource-planning problems we focus on here, such a high level of process detail is unnecessary.

From our literature review, we find that highly detailed surgical process data has been used for simulation studies - either to generate input for surgery schedule optimization models [4, 67] or to investigate the relationships between system parameters and their impact on the OR performance [61]. The effect of different statistical methods for process duration prediction on the OR performance has been analyzed as well [49]. The data is also used for detailed modeling of perioperative resources and their process step-specific allocation with project-scheduling-related [74] or flow-shop-related approaches [51]. With detailed process data, overlapping processes can be modeled, which is, for example, directly being used by the research on OR process design [5, 12, 40]. A high level of process detail is generally not necessary for other typical OR planning problems on the strategic or tactical level, such as dimensioning and allocating OR resources [37]. Detailed process data could, however, be used for the strategic problem of layout planning [63], where the focus lies on the pathways of the different stakeholders in the OR. On the operational level of planning, detailed process modeling enables more realistic modeling in general [5], and individual duration modeling for each process step, e.g., distribution fitting [67].

As mentioned, in less detailed surgical process data, surgeries are usually considered as a whole, i.e., with only one process step. Then benchmarking sets with realized surgery durations per surgery type [36] or fitted theoretical distributions per type together with fixed capacity allocation decisions and case mixes can be used for surgery scheduling. Here, surgery scheduling on the operational level is usually divided into advance scheduling, i.e., surgical cases are assigned to an operating room on a specific day, and allocation, i.e., sequencing of the surgeries, potentially assigning start times. In addition, that data may be used for rescheduling, e.g., if elective surgeries have to be postponed due to arriving emergencies. For example, Jung et al. [44] present optimization models for advance and allocation scheduling as well as a rescheduling procedure. Dexter and Traub [18] investigates surgery scheduling heuristics via simulation, and Landa et al. [50] consider advance and allocation scheduling with stochastic surgery durations, also using waiting list data.

When going to higher levels of planning, less detailed data is usually used. On the tactical level, operating room capacity is allocated to different patient groups, e.g., through block scheduling and fixing a master surgical schedule. This is often done on the level of specialties. Further, staffing and rostering decisions for operating room staff are usually based on the master surgical schedule [8, 23]. Vanberkel et al. [92] relate the master surgical schedule to the resulting capacity usage of downstream resources such as ward beds. Here, for every specialty, they assume a distribution over the number of surgeries that can be performed in a surgery session. Jung et al. [44] allocate capacity for elective surgeries such that emergency patients can also be treated. To this end, they classify surgeries into short, medium, and long surgeries.

Finally, on the strategic level of planning, surgical process data can support service design, case mix, and capacity dimensioning decisions. On this level, models usually assume deterministic values, such as demand volume or required capacity per patient type [39]. In addition, information is needed on costs and profits for serving certain patient types. For example, Blake and Carter [10] propose a goal programming approach to decide on the case mix and volume for physicians using deterministic values for needed surgery and ward capacity per patient type.

### Benchmark sets

As we present a new data collection in Section 3.2 and discuss its usage potential for OR planning research, we are interested in how surgical process data like ours can be made ready for use by fellow researchers. We use the work by Leeftink and Hans [53] as guidance for preparing the data so that benchmark sets can be derived from it. Benchmark sets are crucial for performance comparison of solution approaches on different problem scenarios since not all methods perform equally well in all situations [53]. Some studies provide benchmark sets for general flow or job shop problems [17] or present generic problem instance generators [87]. Most studies on OR planning, such as [50], define their own instance sets. Some make them publicly available, like Riise et al. [74, 81].

Leeftink and Hans [53] focus specifically on generating benchmark sets for surgery scheduling problems. They propose that a surgery scheduling instance should be defined by a surgical case mix and distribution parameters for each type of surgery in the case mix, including expected surgery duration and variation. Note that a surgery in a problem instance, as described by Leeftink and Hans [53], is seen in its entirety, without being divided into separate process steps. The authors suggest an approach for characterizing the case mix of a problem instance and generating several surgery scheduling instances, theoretical and based on real-life data from five different Dutch hospitals. Like Riise et al. [74], they make their benchmark sets publicly available [88]. Several studies have already used these benchmark sets since [38]. Leeftink and Hans [53] conclude their work by suggesting a method for determining the proximity of problem instances in a particular benchmark set and subsequent selection of the least similar instances to ensure the required diversity of the benchmark set.

### Surgical process data benchmarking initiatives

We previously mentioned that surgical process data could be used for benchmarking purposes. Since we present a benchmarking program established by professional associations from Germany, Austria, and Switzerland in Section 3, we shortly list similar initiatives from other countries.

Surprisingly, we did not find many examples of national OR benchmarking initiatives. We start by naming two further German initiatives similar to the one we focus on in this study. One is the benchmarking initiative by Krankenhauszweckverband Rheinland with 87 participating hospitals in 2020 [46]. The other is administrated by BInovis GmbH and JR Consulting oHG and claims its unique approach by evaluating organizational aspects of an OR, additionally to typical process KPIs [43]. For English-speaking countries, we find evidence that in 2011, 471 hospitals and ambulatory surgery centers from the USA, Canada, Saudi Arabia, Australia, and New Zealand participated in the so-called “OR Benchmarks Collaborative,” run by McKesson Enterprise Intelligence, USA [25]. Boggs et al. [11] update the Procedural Times Glossary (PTG) of the US Association of Anesthesia Clinical Directors and note that the PTG has already facilitated benchmarking initiatives. Unfortunately, the authors do not name any examples. Similarly to the PTG, operating theatre efficiency guidelines exist in Australia [1, 82]. We found evidence that the National Health Service (NHS) England, specifically the NHS Benchmarking Network, reports annual benchmarking results in its “Operating Theatres Project” [66], with 69 hospitals participating in 2018 [65]. The results include insights on OR performance indicators such as utilization or turnaround time [65, 66].

We found only one national benchmarking initiative from a non-German-speaking country that we consider similar to the one we focus on in this paper: The benchmarking program of the university hospitals in the Netherlands, established in 2005. The surgical process data of the seven participating clinics are processed and analyzed centrally. The hospitals regularly receive insights on the efficiency and profitability of their ORs compared to fellow benchmarking participants. The participating clinics are encouraged to exchange best practices with each other. The collected data can be provided in anonymized form for scientific studies. The level of detail in the data is high, with several time stamps corresponding to the surgical and anesthetic procedures collected per surgery [90, 91].

## The OR benchmarking program surgical process data and its potential for OR planning research

### The OR benchmarking program of German-speaking countries

#### The German Perioperative Procedural Time Glossary

In 2008, the first version of the “The German Perioperative Procedural Time Glossary” (GPPTG) was published, following the emerging demand for a standardized, KPI-based OR management and external benchmarking among German hospitals [9]. The Glossary was the product of a joint effort by the German professional associations of anesthetists (BDA), surgeons (BDC), and OR managers (VOPM). The GPPTG has been revised and updated twice since - in 2016 and 2020. In the 2020 version, the Austrian and Swiss associations of OR managers (VOPMÖ and SFOPM, respectively) became involved as well, extending the validity of the GPPTG to all three German-speaking countries. In its most recent version, the Glossary contains 41 defined perioperative process time points, categorized into subcategories patient logistics, OR logistics, anesthesia, and operation. Surgical process steps based on these time points and typical KPIs concerning the OR performance are also defined. However, the time points or the process steps do not suggest a “standard” surgery process but rather encompass typical milestones of a generic surgical process. The entire perioperative process is covered, from the patient being called to the patient being discharged from the PACU. However, the GPPTG focuses on the patient’s path through the OR, so other OR-related tasks, such as documentation or planning, are not included [7].

#### Benchmarking program

In connection with the initial publication of the GPPTG, the aforementioned benchmarking program for surgical process data was established in 2009. From the outset, its central purpose has been to provide participating hospitals with an opportunity to compare OR performance among each other and, with this, to evaluate one’s potential for improvement. The technical implementation is carried out by a neutral party company (digmed GmbH, Hamburg, Germany). A participating hospital typically submits all its routinely recorded OR process data monthly. The data collection itself must follow the GPPTG. Participation in the benchmarking is possible by submitting at least two time stamps per surgery: Incision and closure. Additional required information for each surgery involves the date, the surgical department, the operating room, and the unique (anonymized) identification of the operated patient [9]. In principle, participation in the benchmarking program is open to any German, Swiss, or Austrian hospital. However, the benchmarking results are provided to the benchmarking participants only, except for scientific studies. Anonymized data can be provided for the latter [9], and there are already studies that use the benchmarking data for research on OR performance [21].

The number of hospitals participating in the program has grown from 20 hospitals in 2009 [9] to over 320 German, Austrian, and Swiss clinics today [20]. Among the hospitals, all levels of care (LOC) are represented [9, 20].^2^

### Surgical process data from the OR benchmarking initiative

#### The *2019 data set*

A data set from the previously described benchmarking program was kindly provided to us by digmed GmbH. We use this data to derive process durations and case mix distributions. The data set includes all surgical data for 2019 and all participating German hospitals (Austrian and Swiss hospitals were not included). The effect of the COVID-19 pandemic on the OR operations in German hospitals and thus on the corresponding data in years starting 2020 is non-neglectable, so the 2019 benchmarking data represents the latest non-COVID-affected situation of German ORs. In the data set, which we call the *2019 data set*, 212 hospitals are represented in total, which accounts for around 11% of all German hospitals [83]. The *2019 data set* consists of 2,035,126 data points, i.e., recorded surgeries.^3^ For each surgery, the unique hospital ID, the hospital’s federal state, the hospital LOC, the surgical specialty,^4^ the surgery date, the OR, and the unique ID of the patient’s hospital stay are recorded. Further surgery-specific parameters are optional and not always recorded for all data points or by all hospitals. Those parameters include the main OPS^5^ code of the operation, the anesthesia type (local or not local, i.e., general anesthesia), the type of surgical patient (inpatient or outpatient), the urgency (elective or corresponding to a particular level of emergency, following the GPPTG classification [7]), the main operating hours of the corresponding surgical specialty (K18a [7]) and the size of the OR block capacity assigned to the surgical specialty in the particular OR and on the particular date (K18 [7]). In Table 1, all GPPTG times stamps included in the *2019 data set* are listed.^6^ Table 2 includes all process times, which can be calculated using these time stamps as defined by the GPPTG.

**Table 1:** GPPTG times stamps included in the *2019 data set*

Time stamp code	$\text {Time stamp}^a$
P2	Patient Arrival at OR suite
P5	Patient In OR
P7	Patient Out of OR
P8c	Start PACU
P10	End OR Cleaning
A6	Start Anesthesia
A7	Anesthesia Ready
A9	End Anesthesia
O8	Incision
O10	Closure
O11	End Follow-up Surgical Measures

^a^The English terms are taken from GPPTG 2020, although in 2019 the previous 2016 version was still valid. The latter, however, had not been translated into English

**Table 2:** Process times based on the available GPPTG time stamps in the *2019 data set*

Process stamp code	$\text {Process stamp}^{b}$
K2	Anesthesia Induction Time (A6 to A7)
K3	Anesthesia Emergence Time (O11 to A9)
K7	Surgical Lead-in (A7 to O8, or P5 to O8, if P5 after A7)
K8	Incision-to-Closure Time (O8 to O10)
K9	Surgical Lead-out (O10 to O11)
K10	Perioperative Time (A7 to O11, or P5 to O11 if no anesthesia used)
K13	Net Anesthesia Time (A6 to A9)
K15b	Turnover Time Anesthesia (O11 to A7 of the following case)
K16	Closure-to-Incision Time (O10 to O8 of the following session)
K17	Column Time (P5 to P7)
K17a	Room Occupied Time (P5 to P10)

^b^The English terms are taken from GPPTG 2020, although in 2019 the previous 2016 version was still valid. The latter, however, had not been translated into English

digmed GmbH conducts data plausibility checks to ensure high data quality [9]. The latter is required for benchmarking analyses and scientific studies [45, 78, 79]. It should be noted that hospitals that join the benchmarking program tend to improve the quality of the recorded surgical process data (sometimes remarkably) over time [9]. In our *2019 data set*, data points are marked if they have passed the plausibility checks by digmed GmbH. These plausible data points account for more than 98% of the data set. We additionally check how well the optional surgery parameters and the procedural time stamps are documented. In Table 3 for each surgery parameter, the percentage of data points with a definite entry, i.e., a recorded value excluding the unknown values, in the total data set are listed. In Table 4, for every time stamp, we list the percentage of the entire *2019 data set* that has the time stamp recorded and is at the same time marked plausible by digmed GmbH.

**Table 3:** Percentage of data points in the *2019 data set* with a definite entry per surgery parameter

$\text {{Surgery parameter}}^c$	Percentage of data points with a definite value in the *2019* *data set*
(Main) OPS code	89%
Anesthesia type	60%
Type of surgical patient	90%
Urgency	83%
Main operating hours of the surgical specialty	92%

^c^We do not consider the block capacity here since a missing value, in this case, does not necessarily indicate missing data but could mean that the surgical specialty didn’t have any capacity allocated in this OR on this date

**Table 4:** Percentage of data points from the *2019 data set* with recorded and plausible time stamps

Time stamp	Percentage of data points with time stamp recorded and plausibility check passed in the *2019 data set*
P2	73%
P5	30%
P7	27%
P8c	6%
P10	3%
A6	82%
A7	85%
A9	80%
O8	98% (all plausible data points)
O10	98% (all plausible data points)
O11	91%

**Table 5:** Chosen values per parameter to process the *2019 data set*

Hospital LOC	Surgical specialty	Type of surgical patient
Basic and Regular Care	General Surgery	Inpatient
Specialized Care	Trauma Surgery	Outpatient
University Clinics	Otolaryngology	
Maximum Care, excl. University Clinics	Gynecology and Obstetrics	

#### Data processing

Following Leeftink and Hans [53], one goal is to determine surgical case mixes from the data for different OR settings. We define the latter using specific parameters and group the raw data accordingly during data processing. We use the hospital LOC and the surgical specialty as setting or grouping parameters following the approach described in Section 2. This differentiation is reasonable since hospitals of different LOCs and surgical specialties typically have differing surgery portfolios regarding the procedures performed. The organization, including the processes and the resources, might also differ. We use the (main) OPS code to represent the surgery type. Based on an additional analysis during data processing, we decided to use the type of surgical patient as another setting parameter. Table 5 shows the values we choose from the data for each parameter. The urgency does not seem to have a significant additional effect on the process durations.

Regarding the parameter anesthesia type, we only consider surgeries not explicitly marked as carried out in local anesthesia since the group represents less than 1% of our final data selection. See Appendix A for more details on our data selection procedure. To determine the case mix for each combination of hospital LOC, surgical specialty, and type of surgical patient, we determine the OPS codes represented in the corresponding data selection and their relative frequency in the considered class.

**Fig. 1:**
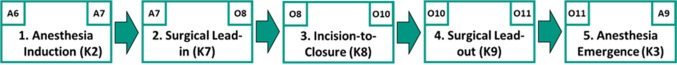
Process steps of a generic surgery according to the process stamps defined in Table 2

**Fig. 2:**
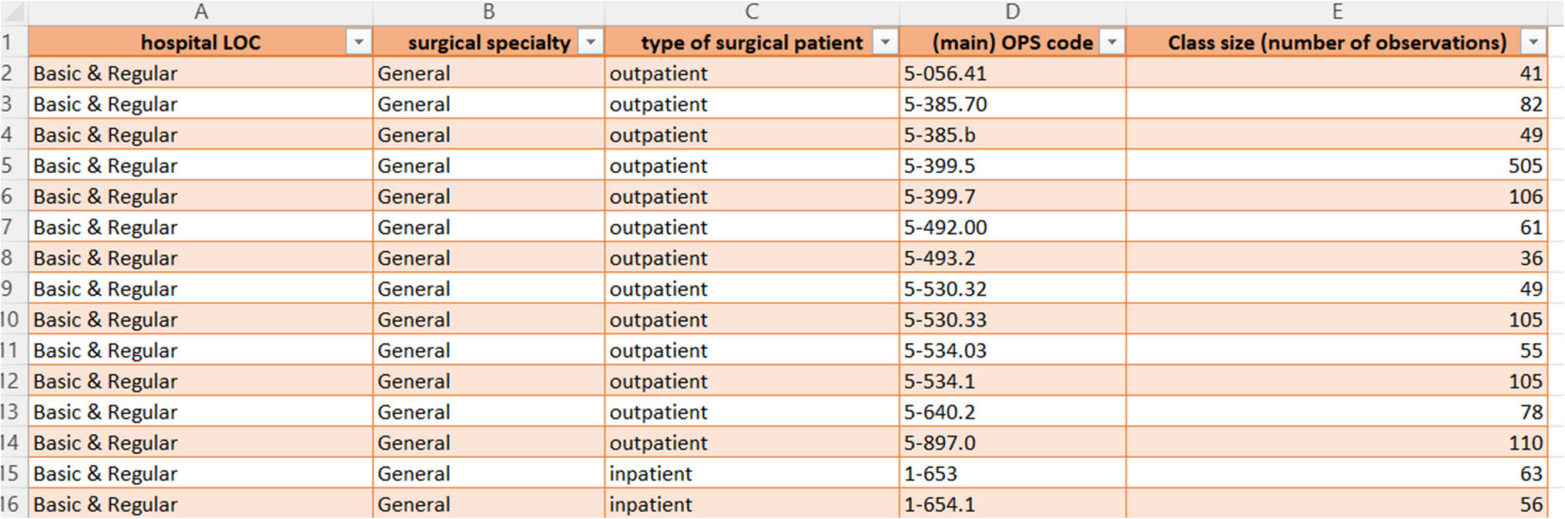
An excerpt from our final case mix collection

For each surgery type in a particular case mix, we fit theoretical distributions (two-parameter lognormal, gamma, and Weibull) for process durations based on the historical data, similar to Leeftink and Hans [53]. Other than Leeftink and Hans [53], we aim for a more detailed modeling of the surgical process than considering a surgery in its entirety. We choose the process-oriented perspective based on available time stamps, focusing on the main perioperative activities, i.e., anesthesia and surgical procedures. We determine the most detailed and consecutive process modeling possible and choose K2, K3, K7, K8, and K9 as our main process steps or times. We assume the process steps to represent a generic surgery as shown in Fig. 1. We note here that this assumed sequence of process steps and time stamps is just one of many possibilities for how the surgical process in a German hospital could be modeled or implemented in reality. As indicated in Table 2, mentioned in 3.1.1 and depicted in detail in Bauer et al. [7] there are different possibilities for the process design of OR logistics, concerning for example the anesthesia procedure or the patient logistics, which can result in different process times definitions in terms of surgical time stamps. We additionally fit distributions for the OR cleaning time (which we define as A9 to P10), so this process step could be modeled separately if desired. Finally, we fit distributions for the closure-to-incision time (K16), although not recommended but still used by some German hospitals proxy for the turnover time [77]. See Appendix B for more details on our distribution fitting method. For our final collection of the process duration distributions, we calculate the expected value and variance besides the estimated distribution parameters for each distribution.

#### Our collection of process time distributions and surgical case mixes

The main output of our previously described analysis and processing of the *2019 data set* is our collection of process time distributions and surgical case mixes. The collection of surgical case mixes is represented by a spreadsheet with four parameter columns: Hospital LOC, surgical specialty, type of surgical patient, and (main) OPS code. The represented values for the first three parameters are listed in Table 5. Moreover, 633 unique OPS codes are represented in our case mix collection. There are 1,685 unique combinations for the four parameters in our final case mix collection. The case mix spreadsheet includes the corresponding class size for each unique combination. It is expressed by the number of observations, i.e., data points or unique surgeries, from our *main data set* (see Appendix A). See Fig. 2 for an excerpt of the case mix spreadsheet.

**Table 6:** Description of the case mix collection: Number of unique OPS codes, total number of observations, and average class size for all represented combinations of hospital LOC, surgical specialty, and type of patient

Hospital LOC/Surgical specialty/Type of patient		Nr. of included OPS codes	Total nr. of observations	Avg. class size (SD)
**Basic and Regular Care**				
General Surgery	inpatient	116	16,254	140.12 (344.86)
	outpatient	13	1,382	106.31 (117.94)
Trauma Surgery	inpatient	100	11,660	116.60 (161.18)
	outpatient	25	1,797	71.88 (79.95)
Otolaryngology	inpatient	20	2,546	127.30 (117.23)
	outpatient	12	1,326	110.50 (175.37)
Gyn and Obstetrics	inpatient	55	6,502	118.22 (114.85)
	outpatient	19	3,582	188.53 (254.07)
**Specialized Care**				
General Surgery	inpatient	182	26,974	148.21 (372.57)
	outpatient	22	2,442	111.00 (140.90)
Trauma Surgery	inpatient	127	11,868	93.45 (125.38)
	outpatient	27	2,275	84.26 (64.82)
Otolaryngology	inpatient	75	9,247	123.29 (191.72)
	outpatient	8	1,272	159.00 (218.95)
Gyn and Obstetrics	inpatient	80	13,464	168.30 (201.02)
	outpatient	25	6,315	252.60 (363.58)
**University Clinics**				
General Surgery	inpatient	113	10,617	93.96 (101.10)
Trauma Surgery	inpatient	78	5,634	72.23 (59.19)
	outpatient	4	139	34.75 (3.11)
Otolaryngology	inpatient	118	15,044	127.49 (155.07)
	outpatient	4	609	152.25 (125.58)
Gyn and Obstetrics	inpatient	42	4,269	101.64 (80.26)
	outpatient	9	799	88.78 (51.34)
**Maximum Care excluding University Clinics**				
General Surgery	inpatient	130	16,094	123.80 (240.15)
	outpatient	9	858	95.33 (44.83)
Trauma Surgery	inpatient	81	7,960	98.27 (103.41)
	outpatient	10	599	59.90 (29.35)
Otolaryngology	inpatient	100	14,158	141.58 (213.16)
	outpatient	13	2,180	167.69 (240.56)
Gyn and Obstetrics	inpatient	53	7,598	143.36 (135.94)
	outpatient	15	2,171	144.73 (169.73)

Table 6 shows for each unique combination of hospital LOC, surgical specialty, and type of patient that is represented in our case mix collection, the number of included OPS codes that correspond with that particular parameter combination, the total number of observations summed up over all these OPS codes, the average class size for the individual OPS codes as well as the standard deviation of the class size. It can be observed that all combinations except for the combination of University Clinics, General Surgery, and Outpatient are represented in our case mix collection. The inpatient combinations typically include a much larger total number of observations and OPS codes than their outpatient counterparts. The number of included OPS codes per combination ranges between 4 (University Clinics, Trauma Surgery, Outpatient) and 182 (Specialized Care, General Surgery, Inpatient). Table 7 shows the five largest OPS codes represented in the case mix collection as measured by the total number of observations. The OPS code 5-511.11 (*“Operations on gallbladder and bile ducts: Cholecystectomy: Simple, laparoscopic: Without laparoscopic inspection of the bile ducts”*) is by far the most prominent with 10,778 observations.

**Fig. 3:**
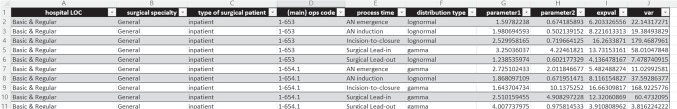
An excerpt from our final main process times distributions collection

The distribution parameters for our five primary process times (see Fig. 1) are listed in another spreadsheet. Here, we again have the four columns corresponding to the previously mentioned parameters. For each of the 1,685 unique parameter combinations, five distributions are included - one for each of the process times. Each distribution includes the distribution type (lognormal, gamma or Weibull), two fitted distribution parameters, and the expected value and variance of the distribution, calculated using the fitted parameters. See Fig. 3 for an excerpt of the main process times distributions spreadsheet.

For the OR cleaning duration and the closure-to-incision duration, we include one individual spreadsheet per process time for the fitted distributions since for OR cleaning, we only use the parameters hospital LOC, surgical specialty, and type of surgical patient (see Table 8) and for closure-to-incision, only hospital LOC and surgical specialty are used, as described in Appendix A. Both spreadsheets are otherwise structured similarly to the spreadsheet with the five main process times distributions.

For all seven considered process times, Table 9 shows the range of the expected values of the fitted distributions expressed by the minimum and the maximum values. The spread is the largest for the incision-to-closure time.

We include Fig. 9 in Appendix B to demonstrate how often each of the three distribution types (lognormal, gamma, Weibull) is represented in our distribution collection for each of the process times.

**Table 7:** The 5 largest OPS codes represented in the final case mix collection, measured in number of observations

OPS-Code	Nr. of observations
5-511.11	10,778
1-672	5,192
5-285.0	4,801
5-530.31	4,676
5-399.5	3,798

**Table 8:** Represented parameter combinations for OR cleaning in the collection of process time distributions

Hospital LOC	Surgical Specialty	Type of patient
Basic & Regular Care	General Surgery	inpatient
		outpatient
	Trauma Surgery	inpatient
		outpatient
	Gyn and Obstetrics	inpatient
		outpatient
Specialized Care	General Surgery	inpatient
		outpatient
	Trauma Surgery	inpatient
		outpatient
	Otolaryngology	inpatient
		outpatient
	Gyn and Obstetrics	inpatient
		outpatient
University Clinics	General Surgery	inpatient
	Otolaryngology	inpatient
	Gyn and Obstetrics	inpatient

With the case mixes, i.e., the empirical distributions of surgery types, and the corresponding distributions of process durations, benchmark sets can be generated as in Leeftink and Hans [53] by choosing a particular value for each of the three setting parameters. Choosing several values simultaneously, e.g., for surgical specialty or type of surgical patient, is also conceivable. In the next section, we plot different exemplary case mixes similarly to Leeftink and Hans [53].

### Discussion

#### Benefits and potential

The surgical process database from the benchmarking initiative described above is a rare example of highly standardized and high-quantity real-world data systematically collected from a large number of data providers. Besides the benchmarking purpose, such a large data set has enormous potential for scientific research. Not only are the recorded process time stamps standardized, following official guidelines of the professional unions involved, but the data also shows an overall high level of quality. Moreover, it has a relatively high level of detail regarding the number of recorded time stamps and other surgery parameters collected per surgery. An obvious advantage of this data source is that it is growing continuously, receiving new data not only from participating hospitals every year but also from new hospitals that join the benchmarking initiative. And since several hundred German hospitals are already participating in this largest national OR benchmarking initiative, we can assume that the data has a reasonably high level of representativeness. However, as we elaborate further below, there is still room for improvement. A continuing expansion of the program in Austria and Switzerland in the coming years is to be expected. Further, the contents and methods of the benchmarking are continuously being improved and extended, as is the underlying process of collecting and processing the data.

**Table 9:** The min-max range of the expected values of the fitted distributions for each considered process time (in min)

Process time	Minimum expected value	Maximum expected value
Anesthesia induction	1.88	62.72
Surgical lead-in	1.83	52.81
Incision-to-closure	6.36	395.78
Surgical lead-out	1.11	20.63
Anesthesia emergence	1.66	26.02
OR cleaning	6.89	15.38
Closure-to-incision	36.52	77.16

**Fig. 4:**
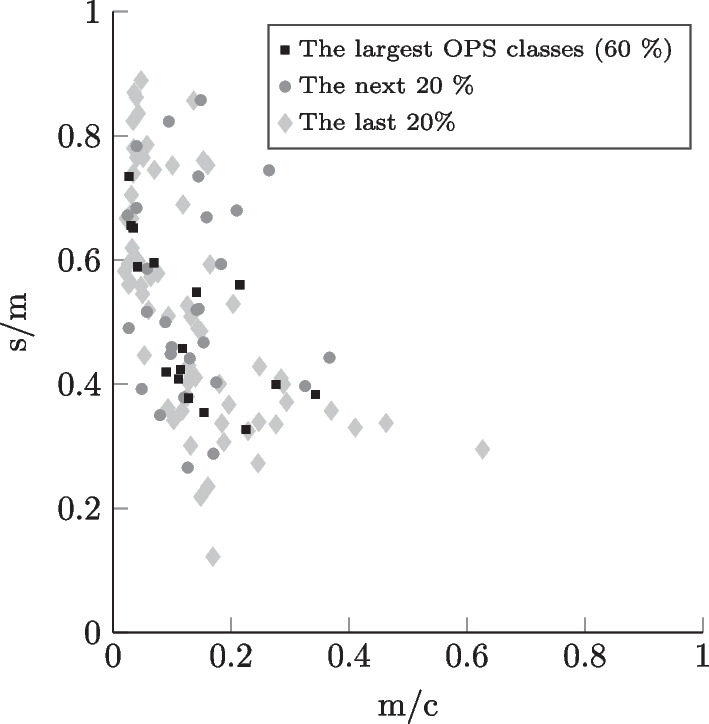
Incision-to-closure time profiles for the case mix corresponding to general surgery inpatients in a hospital offering basic and regular care

**Fig. 5:**
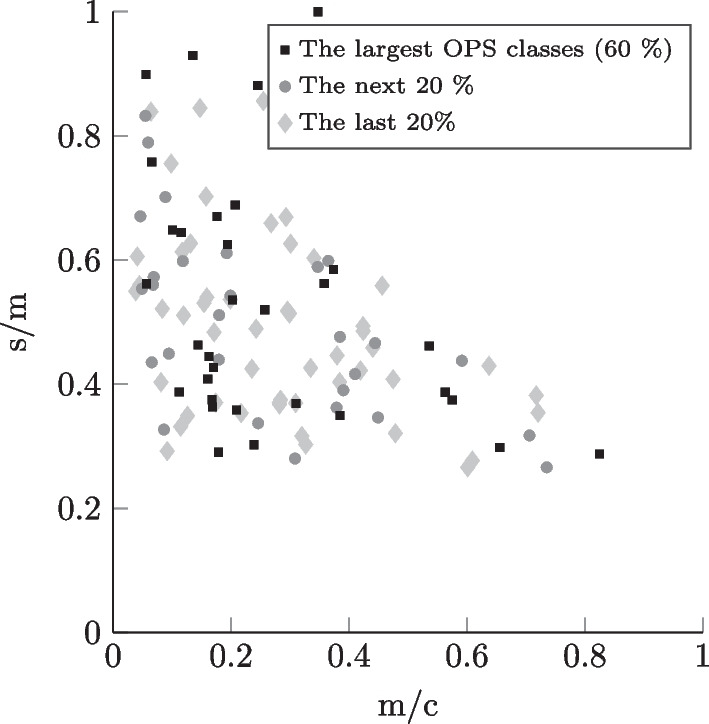
Incision-to-closure time profiles for the case mix corresponding to general surgery inpatients in a university clinic

**Fig. 6:**
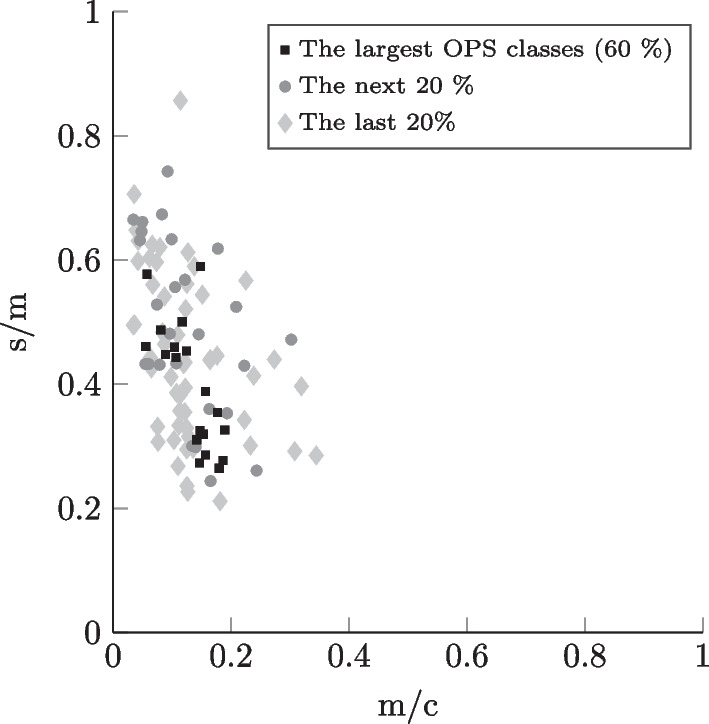
Incision-to-closure time profiles for the case mix corresponding to trauma surgery inpatients in a hospital offering basic and regular care

**Fig. 7:**
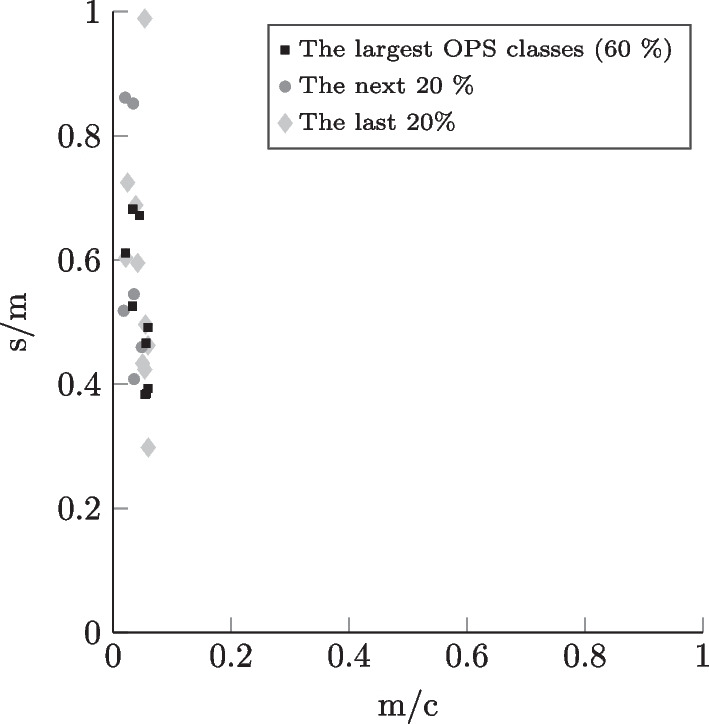
Incision-to-closure time profiles for the case mix corresponding to trauma surgery outpatients in a hospital offering basic and regular care

In the context of research on OR planning, a high number of different OR settings can be modeled - using the data collection that we provide - by choosing either unique combinations of hospital LOC, surgical specialty, and type of surgical patient or by considering, for example, several specialties or both types of surgical patient (inpatient and outpatient) simultaneously. The differentiation by the chosen setting parameters enables more realistic and precise modeling since the surgical portfolios corresponding to the settings differ significantly in practice, as mentioned in Section 3.2. In Figs. 4, 5, 6, and 7, similarly to Leeftink and Hans [53], we plot surgery type profiles for a few exemplary case mixes to demonstrate this issue in terms of procedure duration and its variation. The depicted process step is the incision-to-closure time. The x-axis represents the expected process duration (m) in relation to a typical operating room block duration (c) of 8 hours (480 min). The y-axis represents the coefficient of variation, i.e., the standard deviation (s) divided by the expected duration (m) for the same process time. In the visualization, we have included the case mix distribution of the individual surgery types, i.e., OPS codes: The square dots depict the largest OPS classes cumulatively representing at least 60% of the corresponding case mix. The diamond dots represent the following 20% of the case mix, while the round dots depict the smallest OPS classes in the respective case mix, which make up the last 20%.

We observe, for example, in Figs. 4 and 6 that the outliers regarding the procedure length and variability represent relatively rare surgery types in the respective case mixes. Considering the hospital LOC, we notice when comparing Figs. 4 and 5 that in the case of General Surgery and inpatients, the university clinics show a much more diversified surgical portfolio than hospitals of basic and regular care: Both case mixes include a similar number of OPS codes (116 for basic and regular care, 113 for university clinics), however, with basic and regular care the 60% of the case mix’ volume is represented by the 15 largest procedure types, whereas for university clinics it takes 31 OPS codes to constitute the 60%. We also observe that the incision-to-closure time at university clinics tends to be longer and more variable. Regarding the surgical specialty, we observe by comparing Fig. 4 with the plot in Fig. 6 that the incision-to-closure time of general surgeries tends to be longer and more variable than that of trauma surgery interventions. Finally, the characteristic difference in procedure length between outpatient and inpatient surgeries [14] can be observed when comparing Figs. 6 and 7. We hypothesize that surgery planning approaches will perform significantly differently depending on the hospital LOC, surgical specialty, or type of surgical patient due to the differences in the case mixes.

By choosing a particular combination of parameter values, diverse problem instances, and benchmark sets can be generated from our data collection using the procedure described by Leeftink and Hans [53]. Alternatively, the case mixes and duration distributions of particular OR settings can be used directly, e.g., as input for a simulation model, without generating a finite problem instance. However, when creating benchmark sets, these could be extended by further problem-specific parameters, e.g., urgency or resource-related aspects, as suggested by Leeftink and Hans [53]. According to Leeftink and Hans [53], who refer to Vanhoucke and Maenhout [93], benchmark sets based on our data collection would thus satisfy the condition of extendibility. They would also satisfy the condition of realism since our data collection is entirely derived from real-world data. We argue that these benchmark sets would thus be suitable for the analysis of real-world problems, and the results of such analysis should have higher explanatory power for the corresponding real-world contexts than benchmark sets derived from artificially generated data. (The other two required conditions for a benchmark set, as mentioned by Leeftink and Hans [53], size and diversity, are to be controlled for when a particular benchmark set based on our data collection is actually being generated.)

Compared to the benchmark sets by Leeftink and Hans [53], who consider a surgery in its entirety, the potential benchmark sets based on our collection of surgical case mixes, and process duration distributions would have a higher level of process detail, as we divide a surgery into five process steps and consider the respective durations individually. We argue that only by dividing the surgery process into several process steps can we achieve a more realistic depiction of the OR operations in OR planning models as described in Section 2. Process-specific resource allocation, overlapping processes modeling, and more precise duration prediction are the main advantages of a detailed process modeling approach. As depicted in Section 2, this is particularly interesting for operational planning approaches, e.g., in the context of Job Shop models [69, 74].

A high level of detail is generally desirable for simulative approaches. Moreover, with simulation, it is manageable in terms of computational effort. The benchmark sets based on our collection of surgical case mixes and process duration distributions could thus be used, for example, for simulation studies such as Messer et al. [61] or simulation-optimization approaches as described by Ozen et al. [67] or Kougias et al. [49]. As mentioned in Section 2, highly detailed data can also be useful for research on the strategic level, e.g., for investigating different organizational approaches, including the organization of perioperative processes or the design of spatial resources. Further, our provided high-detailed data can be aggregated and extended to produce suitable input for OR planning models on all planning levels. By summing up sampled process durations, we can consider a surgery as a whole. For advance scheduling and allocation scheduling, we would need additional information on waiting lists and the master surgery schedule. For capacity allocation, e.g., to create a master surgery schedule, we would need extra data on the arrival of demand per patient type including emergencies. Further, the data could be connected with data on staff requirements and the length of stay to take the capacity and scheduling of (downstream) resources and staff into account. On the strategic level, again, information on the demand volume would need to be added, as well as information on costs and profits per patient type. Finally, note that even though many models use aggregated data, they are usually evaluated using simulation which requires a more detailed level of data to approximate the performance in reality as well as possible.

The final advantage of our collection of surgical case mixes and process duration distributions is the mentioned high quantity of the underlying benchmarking data, which, combined with our data processing methods, has enabled a high statistical quality of our calculated case mix and process duration distributions. There is a more practice-oriented potential here as well - the high quantity of benchmarking data can be leveraged by OR management practitioners who only have limited data from their own ORs. Especially for the duration prediction of rare surgical procedures, the data of other hospitals could be used as a planning proxy in practice.

#### Limitations

To finish the discussion section, we want to comment on the limitations of the benchmarking data and the collection of surgical case mixes and process duration distributions we provide. To derive the latter, we focused exclusively on the specific OR context of German hospitals. Our provided data collection could nevertheless be used for research on country-specific differences. Focusing on Germany, however, we face a representativeness issue in the original benchmarking data: As mentioned in Section 2.2, only 11% of German hospitals have participated in the program so far. Moreover, the distribution of the participants concerning the hospital size, the federal state, or the LOC, for example, does not accurately represent the actual proportions. Large hospitals and university clinics, in particular, represent a disproportionately large fraction. This can be attributed to the generally higher interest of these hospitals in process efficiency and progressive OR management methods [68], but also the availability of necessary resources [9].

Another limitation of the benchmarking data is missing data. As we show in Section 3.2.1, the optional surgery parameters such as anesthesia type or urgency and process time stamps other than incision and closure are documented to a varying degree. A more elaborate and consistent hospital data recording practice would be desirable. However, a more significant issue is the information on certain parameters that are not yet recorded. This makes investigating particular research questions using the data alone impossible and requires additional assumptions about the missing contexts.

This is particularly the case with the performed procedures during a surgery. Since only one OPS code is available per surgery record, it is unclear whether there were other procedures carried out during the same operation and, if so, which procedures these were in particular. In such a case, it is also unclear based on what criteria the chosen OPS code was determined to be the main procedure by the submitting hospital. It is moreover unclear whether the procedure was carried out during a session that included multiple operations and, if so, whether the respective operations were carried out simultaneously, sequentially, or in parallel [7]. Such information would be desirable for analyses like ours. However, the corresponding data recording practice might be rather challenging to implement. In our case, we must implicitly assume that the recorded OPS code corresponds to the actual main procedure carried out during the surgery and represents the entire surgery.

**Pre-surgical planning**

Another issue we have to deal with considering the OPS code is that in German hospital practice, the OPS codes are identified and assigned post-surgery. They are used mainly for reimbursement purposes [73]. The pre-surgical planning of a surgery in German hospitals is usually done by using general, sometimes hospital-specific procedure terminology, which only in some cases could be unambiguously matched with OPS codes. Since there is no information on planned procedures in the benchmarking data, for the purposes of research on OR planning, it must be assumed that the performed procedure (main OPS code) represents the planned one. In reality, there can be a bias between the two [19] since the exact procedure cannot always be determined in advance [73].

Following the idea of Riekert et al. [73], we investigate how well a more general OPS classification could serve as a proxy for a planned procedure. A complete OPS code, as represented in the benchmarking data set and our resulting collection of case mixes and process duration distributions, contains at most six characters (excluding a hyphen that follows the first character). We use what Riekert et al. [73] call the third level of OPS taxonomy, namely the first four characters of an OPS code to represent an OPS category. An OPS category contains less information than a complete OPS code. Thus, it could be assumed to represent the information available at the planning stage before the surgery takes place.

We demonstrate this idea for one exemplary OPS category. We choose category 5-870 (“Excision and resection of the mamma: Partial (breast-conserving) excision of the mamma and destruction of mamma tissue” [27]). The complete OPS codes in this category are shown in Table 10 for the setting of specialized care, gynecology and obstetrics, and inpatients. As mentioned, a complete OPS code contains more details on the specific procedure than the corresponding general category, e.g., OPS code 5-870.61 stands for “Excision and resection of the mamma: Partial (breast-conserving) excision of the mamma and destruction of mamma tissue: Local destruction: Defect coverage by mobilization and adaptation of up to 25% of the breast tissue (up to 1 quadrant)” [28].

**Table 10:** Mean and variance of the incision-to-closure time and the frequency per OPS category and the corresponding OPS codes (gynecology and obstetrics inpatients in a hospital offering specialized care)

OPS	Mean	Variance	OPS relative frequency in the case mix
5-870	55.0	953.5	100%
5-870.20	37.3	338.9	1.5%
5-870.21	41.3	479.3	1.6%
5-870.60	34.7	368.4	1.4%
5-870.61	57.0	933.0	2.4%
5-870.90	40.2	679.2	11.1%
5-870.91	38.2	459.8	5.0%
5-870.a0	43.9	729.1	10.5%
5-870.a1	55.1	721.0	38.5%
5-870.a2	60.2	743.9	20.6%
5-870.a3	89.4	1607.1	4.4%
5-870.a5	97.3	1899.3	3.0%

The incision-to-closure time of the OPS category 5-870 is a mixture distribution of mixture components, here the OPS code distributions. For such distributions, the mean can be calculated as the sum of the means of their mixture components weighted by the mixture weights, i.e., the probability or frequency of seeing the specific mixture component. The variance of the mixture distribution can also be calculated analytically [26]. It is given as the mixture of the component variances plus a non-negative term accounting for the weighted dispersion of the means. In our example, the analytically determined variance for the incision-to-closure time of OPS category 5-870 is 951.2, i.e., the sum of 774.5 (mixture of the variances) and 176.7 (the term that accounts for the dispersion of the mixture means), which is very close to the empirical variance that can be seen in Table 10. The slight deviation is due to rounding. As expected, we observe that the dispersion of the mixture means results in additional variability to account for when planning. Even though we can easily determine the moments of mixture distributions, in general, the distribution will not be lognormal, gamma, or Weibull given that the mixture components were of those types. It can even be multi-model.

If one wanted to take on our suggested approach of using OPS categories instead of complete OPS codes for a planning model, then one could use Monte Carlo simulation to generate realized process durations on the OPS category level.

**Missing context**

Besides the planned procedures, the OR planning methods deployed by the hospitals are unknown. We also have no information on the actual process design, including opening hours, of each corresponding OR included in the original benchmarking data set or how it might have changed over time. Further, since we do not have data on cancelations or reschedulings, we cannot accurately depict the actual surgical demand. We derive the case mix of the demand for different surgery types based on the realized surgeries. This realized demand most likely does not represent the actual external demand and is furthermore determined by the OR capacity of the respective hospital. Since process delays, waiting times, and transportation times are not explicitly submitted, there is no possibility of modeling these aspects based on the data. On the other hand, we must assume these artifacts are implicitly included in the process times we have derived from the data.

The process steps for which we provide the duration distributions do not enclose the entire perioperative process. This is, on the one hand, due to the current state of the data collection practice in the hospitals since, as mentioned above, only a few of the available GPPTG time stamps are collected by a significant number of clinics. Also, not all processes, such as OR planning or documentation, are explicitly depicted in GPPTG, as mentioned in Section 3.1. Finally, in the context of the data available to us and this study, we focused only on the main perioperative activities, starting with anesthesia induction and ending with anesthesia emergence.

Our data has no information on OR resources associated with each process step, such as OR personnel, equipment, rooms, or need for downstream resources such as a bed in the ICU or the ward. Considering the OR personnel, it can be assumed that there will not be any detailed information collected as part of the benchmarking program any time soon since individuals-related data has generally not been collected or evaluated in German hospitals so far [6].

Regarding the spatial resources, we have investigated, using the data, whether all the process steps we considered can be assumed to be carried out in the OR itself. For the anesthesia induction, we observe in 41% of the cases that it is finished after the patient enters the operating room (A7 after P5, for all data points in the *main data set* with both time stamps recorded). In the remaining 59% of the cases, the induction is finished before or with the patient’s OR entry, corresponding with the common practice of German hospitals when an anesthesia induction room is used. It is spatially separated from but typically directly connected with the OR. For the anesthesia emergence, in 73% of the cases, the process step is finished inside the OR (P7 after A9).

We investigated the OR cleaning process step similarly. We have found that the cleaning between two consecutive surgeries in the same OR is finished before the anesthesia induction of the latter surgery is completed (P10 of surgery 1 is before A7 of surgery 2) in 92% of the identified turnovers. The average cleaning duration in our final data selection was 12.5 minutes, while the average anesthesia induction was 13 minutes. These findings suggest that the cleaning typically occurs parallel to the anesthesia induction of the following patient. Moreover, it does not necessarily have to be modeled as a separate process step since it typically lasts shorter than anesthesia induction.

**Data selection process**

Since we did not differentiate the data based on additional parameters other than those we chose, our final data selection based on the original *2019 data set* has an implicit issue of heterogeneity. For each combination of hospital LOC, surgical specialty, type of surgical patient, and OPS code, we aggregate across multiple hospitals, anesthesia procedures, urgency levels, surgeons, and other resources and do this for a year. This limits the representativeness of our derived case mixes and distributions. To calculate the latter, we aggregate the benchmarking data across hospitals. Hence, our resulting collection is less suitable for a detailed analysis of one particular hospital and its individual OR operations. Its potential lies thus primarily with a more generic scope of research, although the process duration distributions could be used as a proxy if a hospital’s data is scarce, as mentioned previously.

During our data selection process, we had to meet several more or less arbitrary assumptions, e.g., which process durations we consider implausible. As a result of the data selection, we excluded as much as 90% of the original *2019 data set* to obtain the final *main data set* that we used to derive case mixes and duration distributions. This naturally contributes further to the representativeness issue. The large percentage is mainly due to three major goals of our data selection process: (1) High data plausibility (valid parameter values); (2) exclusion of irregular surgery settings (operating outside regular opening hours, overlapping process steps, local anesthesia procedures); and (3) high level of detail (number of time stamps and grouping parameters). With the latter, we also wanted to ensure a sufficient class size for each unique combination of grouping parameters in the *main data set*. Thus, we only considered the four largest hospital LOCs and the four largest surgical specialties from the *2019 data set* as listed in Table 5 and removed all combinations with class sizes of less than 30 data points in the final data selection. Researchers who wish to use our data collection for their studies should be aware of the fact that it represents these particular OR settings only. See Appendix A for more details on our data selection process.

**Fig. 8:**
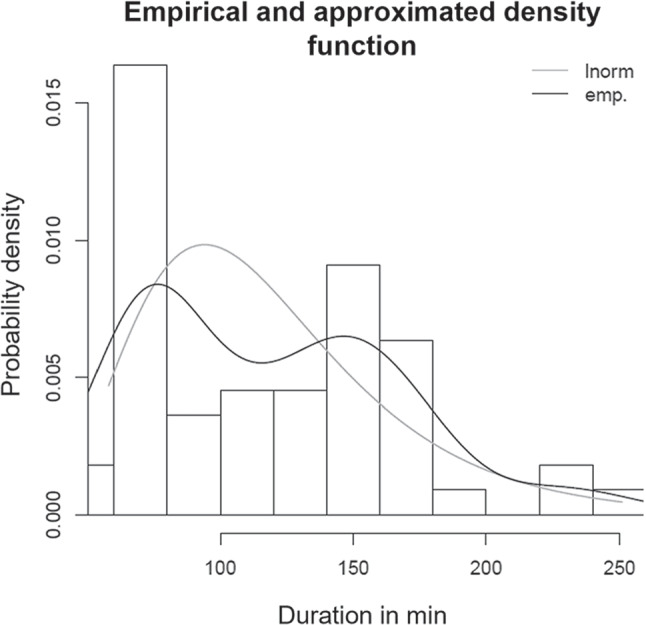
Empirical bimodal and approximated unimodal distribution for the incision-to-closure time of the OPS code 5-059.c7 (otolaryngology inpatients in a university clinic)

When deriving process duration distributions, we encountered goodness-of-fit issues that we had to deal with in every fourth case (see Appendix B). We conclude that the duration distributions we have fitted cannot depict every aspect of the actual surgical process data precisely each time. It is possible that, in some cases, theoretical distributions other than those we considered (gamma, lognormal, Weibull) might have been the better choice. It is also possible that in some cases, an unimodal distribution was not the right approach in the first place, where, for example, a bimodal distribution would represent the empirical data more accurately [56]. Figure 8 shows this in the context of university clinics, otolaryngology, inpatients, OPS code 5-059.c7, and incision-to-closure time.

## Conclusion

To conclude, we summarize the results of our work: We have presented the OR benchmarking initiative of German-speaking countries in the context of research on OR planning for the first time. We elaborated in detail on the properties of the surgical process data collected in the benchmarking program and its potential for OR planning research. Further, we made the processed data freely available, so fellow researchers could use it to test modeling and solution approaches for different OR planning problems. Corresponding to our data selection process, different OR settings determined by the hospital LOC, the surgical specialty, and the type of surgical patient can be investigated using the data collection of surgical case mixes and process duration distributions we provide. Since we break down the perioperative surgical process in several separate steps in our data, it is of particular relevance for highly detailed approaches such as simulation or Job-Shop-like models, especially on the operational level of planning. However, when aggregating and extending our provided data with additional information, it can be used for OR planning problems on all planning levels. Finally, we have discussed the benefits and limitations of the benchmarking program, the collected surgical process data, and our data processing approach and its results. With its vast data collection, we argue that the benchmarking initiative poses a unique opportunity for scientific research on OR operations.

We suggest several directions for further studies and for applying our results in the following. For the researchers who want to use the data collection of surgical case mixes and process duration distributions we provide to generate benchmark sets or problem instances, we recommend using the methods described in Leeftink and Hans [53].

Following our suggestion to use the data of the OR benchmarking initiative of German-speaking countries for the research on OR planning, we encourage fellow researchers to continue to work on and develop planning models and methods that are highly detailed in terms of using high-dimensional input data and modeling the perioperative surgical process with several separate process steps in particular. There are still few such approaches today, which might be because there was not much detailed real-world data available previously.

We further believe that a systematic review article on using real-world surgical process data and the corresponding modeled surgical processes would significantly contribute to Operations Research in OR planning.

We want to suggest several further possibilities for processing surgical process data from the OR benchmarking initiative that we have presented here. First, the original *2019 data set* that we used could be processed and prepared differently, as we did here, in a more suitable way for a particular research purpose. This could, for example, be done by implementing a different data selection approach. Alternatively to our procedure of fitting the duration distributions for separate surgical process steps, the surgical case time, i.e., the duration of a surgery as a whole, could be in the focus. Since distribution fitting might be difficult for potentially multi-modal distributions, we recommend directly working with benchmark sets.

Another research path would be to use data more recent than 2019, for example, to investigate the effect of the COVID-19 pandemic on OR operations. This would also be a possibility to include more data parameters in the analysis. A deeper dive into the data analysis might also be of interest: An approach different from ours could be employed for the distribution fitting of process durations. In some cases, one could test whether theoretical distributions other than lognormal, gamma, or Weibull might fit better. In other cases, multimodal distributions might be a promising approach, as Section 3.3.2 mentions. An ensuing research question would be whether multimodal duration distributions require new, specific planning approaches since existing planning models and methods typically deal with unimodular distributions. Finally, an extensive analysis of different data patterns in the benchmarking data, e.g., dependencies and correlations between individual process times, using elaborate data analysis methods is most likely to generate new valuable insights.

The benchmarking data could become even more valuable for OR decision-making if additional attributes were collected, e.g., information on the setup, such as the underlying master surgical schedule and applied scheduling procedures, the usage of (downstream) resources and staff, and the actual demand for surgery, including waiting lists.

As for the OR benchmarking initiative that we have presented in this paper, we hope to increase awareness of the particular research field of OR planning and the inherent potential of the benchmarking data in this regard. Any initiative facilitating future scientific endeavors in this field, such as automated data processing (e.g., duration distribution fitting) as part of the regular benchmarking operations, would be very welcome.

At the very end, we want to use the final opportunity to address professional associations of surgeons and anesthesiologists, OR managers, and Operations Research scientists in the field of OR planning from other countries and encourage them to pursue OR benchmarking initiatives and leverage the potential of existing projects in a similar way that we did in this study. Specifically, we mean processing surgical process data and providing the results with free access as we did. We think that an international database of surgical process data benchmark sets from different countries could be a very promising endeavor for the entire research field and, thus, for OR operations around the globe.

## Figures and Tables

**Fig. 9:**
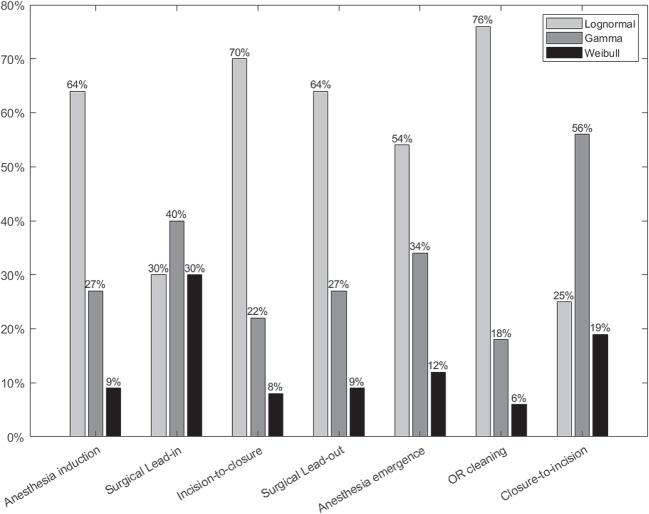
Relative frequency of best fit distribution type for each process time

**Fig. 10:**
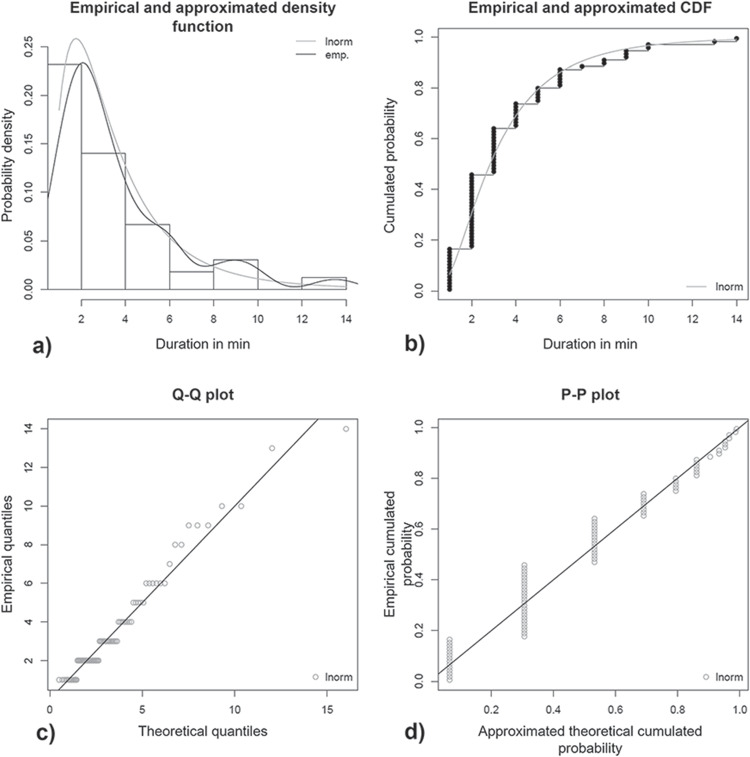
Probability density, cumulative distribution, Q-Q and P-P plots of anesthesia induction for OPS code 5-793.k6 (Trauma surgery inpatients in a hospital offering specialized care)

**Fig. 11:**
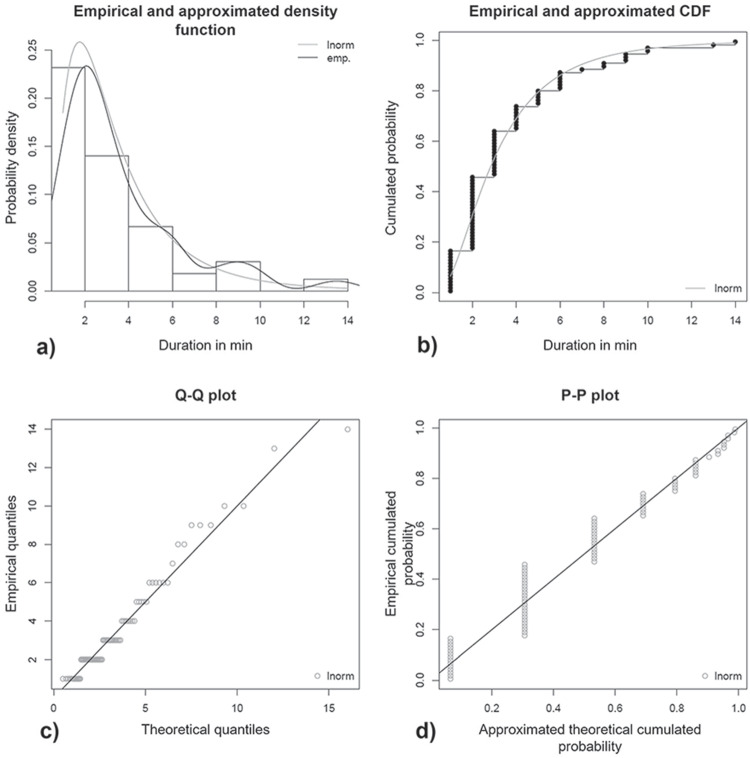
Probability density, cumulative distribution, Q-Q and P-P plots of surgical lead-out for OPS code 5-712.0 (Gynecology and obstetrics inpatients in a university clinic)

**Fig. 12:**
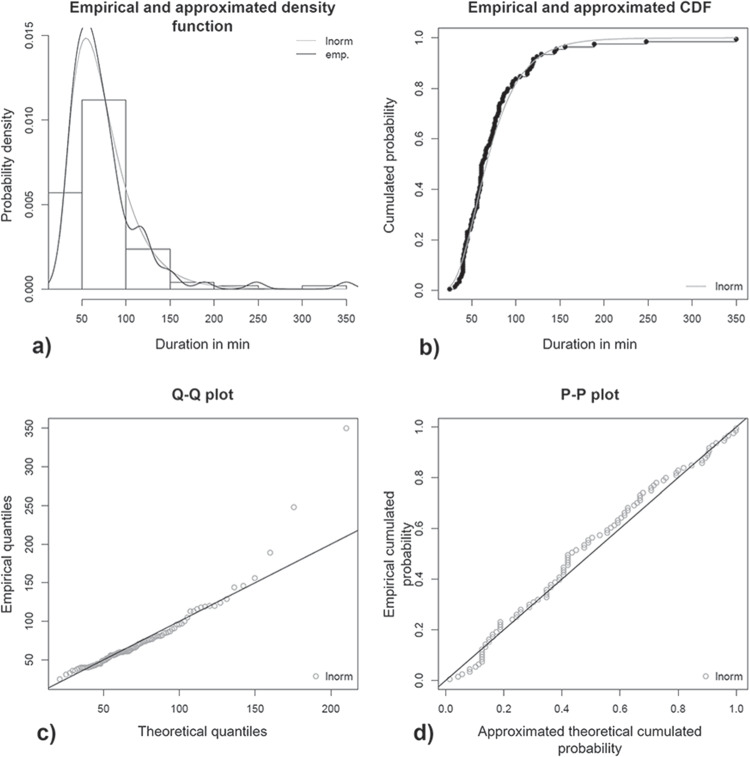
Probability density, cumulative distribution, Q-Q and P-P plots of incision-to-closure time for OPS code 5-465.1 (General surgery inpatients in a hospital offering basic and regular care)

## Data Availability

Surgical case mixes and distributions of peri-operative surgical process durations for German hospitals [data set]. 2022. Zenodo https://doi.org/10.5281/zenodo.7147921

## Appendix A Data selection

We have to exclude the data of one particular hospital from the original benchmarking data set due to incorrect formatting of the processing times. We also exclude all surgeries with incisions taking place outside of 2019. We call the resulting data set the *2019 data set* as described in Section 3.2. We focus only on the regular OR operations. Therefore, we exclude all records of surgeries that took place on weekends or public holidays, as well as surgeries, carried out outside the main operating hours of the respective surgical department or on days for which the department did not have any surgical capacity in the respective OR assigned.

Moreover, we only keep the surgical records that are marked plausible by digmed GmbH, have the main OPS code recorded, the main time stamps we are interested in (A6, A7, A9, O8, O10, O11) recorded, and the corresponding process time (K2, K3, K7, K8, K9) strictly greater zero. Other than Messer [60], we do not allow any process time to be zero. We keep elective as well as urgent surgeries. However, we simplify the classification by grouping all surgeries with an emergency level assigned into one emergency group. We additionally identify consecutive surgeries in the data set, i.e., surgeries that took place in the same hospital, OR, and date. We determine the closure-to-incision time (K16) for each pair of consecutive surgeries, as well as whether the anesthesia start (A6) of the latter surgery took place before the anesthesia end (A9) of the previous surgery. We mark such overlapping surgeries and exclude them from further consideration since process durations of overlapping surgeries correspond to a different process organization and differ significantly compared to the strictly consecutive surgeries, according to Schuster et al. [79]. We assume that all other surgeries in our data set were non-overlapping.

We choose the four largest hospital LOCs in our data set and the four largest surgical specialties as listed in Table 5. We continue by rounding all process times to the nearest minute and carry out our secondary plausibility checks: We filter out all surgery records if either process times anesthesia induction, anesthesia emergence, surgical lead-in, or surgical lead-out lasting longer than 180 minutes. We defined this plausibility check together with digmed GmbH. More detailed plausibility checks would require significantly greater effort and medical expertise, especially for process times that are too short. Note that we allow for all strictly positive incision-to-closure durations. However, we exclude all surgeries with a closure-to-incision time less than or equal to zero, using this secondary process time for an additional plausibility check. We also removed all surgeries with invalid OPS codes and all hospitals with less than 100 data points in the remaining data set. As mentioned in Section 3.2, we exclude all surgeries with local anesthesia explicitly recorded and assume the anesthesia of all remaining surgeries to be not local.

We use one-way ANOVA to determine whether we should use urgency (“elective” vs. “emergency”) or type of surgical patient (“inpatient” vs. “outpatient”) as additional grouping parameters for case mix definition besides hospital LOC and surgical specialty. For this, we randomly choose five different combinations of hospital LOC, surgical specialty, and OPS code (aiming for a sufficiently high amount of data points for each parameter combination) for each of the two parameters and run ANOVA for the anesthesia induction duration and the incision-to-closure time for each of the combinations. 8 out of 10 ANOVAs for the type of surgical patient show a significant impact of the grouping parameter on the considered process duration (alpha=0.05). For urgency, 4 out of 10 ANOVAs show significant results. Based on this, we decided to use the type of surgical patient as a further grouping parameter. Consequently, we removed all surgeries with no type of surgical patient recorded from our data set. We do not further differentiate based on the surgery urgency.

To ensure a sufficient statistical power of the distributions that we later fit individually for every unique combination of hospital LOC, surgical specialty, type of surgical patient, OPS code, and process time, we eventually removed all combinations with class sizes of less than 30 data points.

Our final data selection which we name the *main data set* includes surgical process data from 411 surgical departments of 139 hospitals with a total of 207,635 data points, with 1494 observations per hospital or 505 observations per department on average. Based on our *main data set*, we derive two separate data sets to determine the duration distribution for OR cleaning (calculated as P10 - A9) and closure-to-incision time, respectively. In each of the two additional data sets, we remove all data points with values of less than or equal to zero and greater than 120 min for the corresponding process time, following Schuster et al. [79]. We group the data using hospital LOC and surgical specialty for the closure-to-incision time.^7^ For OR cleaning, we additionally differentiate based on the type of surgical patient after carrying out a corresponding ANOVA, similar to the procedure described above. We again remove classes with less than 30 observations in each data set. Due to our data selection procedure, not all parameter combinations of the three grouping parameters are represented in the final *OR cleaning data set* (see Table 8). The resulting *OR cleaning data set* includes 7,442 data points, and the *closure-to-incision data set* has 120,197 observations.

## Appendix B Distribution fitting for process durations

For each unique combination of selected parameters and for each process time, we fit a (two-parameter) lognormal, a gamma, and a Weibull distribution using maximum-likelihood estimation (MLE) [52] in R [15]. The lognormal distribution is among the most popular distributions for fitting the incision-to-closure time and the entire surgery duration, i.e., case time [84, 86, 98]. We choose these three theoretical distributions since they all are suitable to depict the typical properties of surgical process durations: Continuity, positive skewness, left-side boundedness (by the zero), and right-side unboundedness [60]. We determine the distribution parameters and the corresponding standard errors for each fitted distribution. We calculate the Akaike Information Criterion (AIC) [2] for each of the three corresponding MLE estimations for each parameter combination and process time. We then chose the theoretical distribution with the lowest AIC value as the best fitting [22]. Out of the 8,425 distributions we have fitted, the lognormal distribution was the best fit in 56% of the cases, the gamma distribution in 30% of the cases, and the Weibull distribution in the remaining 14%. Figure 9 shows in how many cases percentage-wise each of the three distribution types was the best fit for each of the process times. We observe that the lognormal distribution was the best fit in most cases for anesthesia induction, anesthesia emergence, incision-to-closure, surgical lead-out and OR cleaning. The gamma distribution was the most common best fit for surgical lead-in and closure-to-incision duration.

In each case, we additionally perform goodness-of-fit tests for the selected best-fit distribution: Kolmogorov-Smirnov (K-S) [52] for either lognormal, gamma, or Weibull and additionally Shapiro-Wilk (S-W) on the logarithmized data if the chosen distribution was lognormal [85]. We additionally analyze some randomly chosen distributions graphically if the p-value of a calculated test statistic is below $\alpha =0,01$. Figure 10a to d show the analyzed plots for the density, the cumulative distribution, the Q-Q, and the P-P plot [52] respectively, for the randomly chosen case of specialized care, trauma surgery, inpatients, OPS code 5-793.k6, and anesthesia induction. The estimated lognormal distribution was rejected in this case due to the S-W test. The deviation between the empirical and the fitted distribution is observable in the Q-Q plot in Fig. 10c and the P-P plot in Fig. 10d. Moreover, in 10a, the aforementioned typical properties of a surgical process duration distribution with the empirical density function can be observed.

Out of 8,425 performed estimations with our *main data set*, the selected fitted distribution was rejected in 24% of the cases by the goodness-of-fit testing. Strum et al. [85] name several potential explanations for the rejection of a fit by a goodness-of-fit test, which hold especially in the case of the lognormal distribution. The latter is over-proportionally often rejected compared to the gamma and the Weibull distributions in our case (38% versus 8% and 3%, respectively). One issue can be large sample sizes [85]: In our case, they range from 30 to 4395 observations per class, with 87% of all classes having a sample size equal to or less than 200. Out of these, 83% have passed the goodness-of-fit test(s). For the remaining 13% of all classes with a sample size greater than 200, 33% of the classes have passed the goodness-of-fit test(s). Another explanation can be the so-called ties [22], i.e., local accumulations of particular discrete values despite the fitted distribution being continuous. In our case, this is especially relevant for typically short process times such as surgical lead-out since the one-minute rounding precision has a substantial effect, in this case, similarly to Strum et al. [85]. Figure 11 displays this issue for the case of university clinics, gynecology and obstetrics, inpatients, OPS code 5-712.0, and surgical lead-out.

Further explanations for rejection by a goodness-of-fit test, as discussed by Strum et al. [85] are data outliers, which can be a possible explanation in our case for the incision-to-closure time, for which we explicitly did not set any upper limit when processing the original data (see Appendix A). This can be, for example, observed for basic and regular care, general surgery, inpatients, and OPS code 5-465.1 in Fig. 12. Since we do not account for every possible grouping parameter, such as anesthesia type or surgeon, when clustering data into classes, our fitting samples are implicitly heterogeneous, which can be another reason for imprecise distribution fitting [85]. Finally, we observe in our analyses of the lognormal distribution that the K-S test acts more conservatively than the S-W test: 41% of all rejected lognormal estimates are rejected by both tests. However, in the remaining 59%, the rejection is only made by the S-W test, while in only 2 cases out of 1,796, the K-S test alone was responsible for the rejection of the fit.

We decide to stick to all of the fitted distributions, even those rejected by the goodness-of-fit test(s), because following Strum et al. [85], we do not rely on the tests alone but use them in combination with graphical analysis, where we find the fits to be sufficiently good, given the discussed peculiarities of the sample data. We carry out the distribution fitting of the OR cleaning time and closure-to-incision time separately but similarly as described above.

## Footnotes

1 As of 2022.

2 Hospital LOC is, in this case, the German classification of acute care clinics based on clinic size (measured in beds) and specialization of the medical offering. Note that the hospital LOC classification in the benchmarking program is partly provisional since there is no standardized classification across all German federal states so far [95]. The terms used in the benchmarking program also correspond only approximately to the according classifications in Austria [29], and Switzerland [13].

3 We use the term surgery synonymously to what in the GPPTG is defined as operation. The latter can consist of one or more procedures. One or more surgeries make up a session [7].

4 Here we use the codes published by the German Hospital Federation (“Deutsche Krankenhausgesellschaft”) and the National Association of Statutory Health Insurance Funds (“GKV Spitzenverband”). The coding approach can differ across federal states [71].

5 The main procedure of the surgery with respect to the “Operationen- und Prozedurenschlüssel,” i.e., Operation and Procedure Code [72], which is the German modification of the International Classification of Procedures in Medicine [24].

6 digmed GmbH had data on other time stamps available; however, those were submitted by only a few hospitals and resulted in too few data points for our purpose.
